# Very-Low-Density Lipoprotein: Complex Particles in Cardiac Energy Metabolism

**DOI:** 10.1155/2011/189876

**Published:** 2011-07-03

**Authors:** You-Guo Niu, Rhys D. Evans

**Affiliations:** ^1^Department of Physiology, Development and Neuroscience, University of Cambridge, Downing Street, Cambridge CB2 3EG, UK; ^2^Department of Physiology, Anatomy and Genetics, University of Oxford, Parks Road Oxford OX1 3PT, UK

## Abstract

The heart is a major consumer of energy and is able to utilise a wide range of substrates including lipids. Nonesterified fatty acids (NEFA) were thought to be a favoured carbon source, but their quantitative contribution is limited because of their relative histotoxicity. Circulating triacylglycerols (TAGs) in the form of chylomicrons (CMs) and very-low-density lipoprotein (VLDL) are an alternative source of fatty acids and are now believed to be important in cardiac metabolism. However, few studies on cardiac utilisation of VLDL have been performed and the role of VLDL in cardiac energy metabolism remains unclear. Hearts utilise VLDL to generate ATP, but the oxidation rate of VLDL-TAG is relatively low under physiological conditions; however, in certain pathological states switching of energy substrates occurs and VLDL may become a major energy source for hearts. We review research regarding myocardial utilisation of VLDL and suggest possible roles of VLDL in cardiac energy metabolism: metabolic regulator and extracardiac energy storage for hearts.

## 1. Introduction

Very-low-density lipoprotein (VLDL) and chylomicrons (CM) comprise the triacylglycerol-(TAG-) rich lipoproteins in the circulation. The assembly and secretion of VLDL particles takes place in parenchymal liver cells. The basic structure of VLDL is such that the hydrophobic triacylglycerol molecules, together with cholesterol ester, form the particle core and are coated by a relatively hydrophilic monolayer consisting of phospholipids, free-cholesterol, and apolipoproteins [1–3]. Apolipoprotein (apo) B100 is a 4536 amino acid glycoprotein, synthesised in the liver and the major structural protein of VLDL. Interestingly, each VLDL particle has just one copy of apo-B100, which means the concentration of apo-B100 represents the number of particles, and this molecule constitutes the basic scaffolding, upon which other compositional components are attached during the assembly process of the lipoprotein. 

The TAG core of the VLDL particle is synthesised by liver cells using different endogenous fatty acid sources. These include fatty acids derived from hydrolysis of adipose tissue TAG stores (lipolysis), fatty acids derived from peripheral hydrolysis of plasma lipoprotein (VLDL and chylomicron) TAG cleared by the liver, and newly synthesised fatty acids via hepatic *de novo* lipogenesis [4]. Therefore, VLDL has been regarded as a transport vehicle responsible for redistribution of endogenous lipids from liver to peripheral tissues. 

But what is the purpose of this redistribution? It appears that the major function of hepatic VLDL secretion is to buffer plasma “free” fatty acid levels through their conversion to core VLDL-TAG [4]. In part, the consequence of this conversion is that energy contained within fatty acid is stored and transported in an alternative (nontoxic) form, the esterified fatty acid-VLDL TAG core. This is of particular importance for the heart since lipids are the major cardiac energy substrates.

Cardiac function is critically dependent on substrate utilisation, and changes in myocardial substrate selection can have a major impact, both positively and negatively, on myocardial contraction [5]. The heart is capable of utilising a wide variety of substrates including carbohydrates, lipids, amino acids, and ketone bodies for its energy requirements, but this utilisation is condition-dependent according to substrate availability and work load. Although amino acids and ketone bodies are energetic substrates for heart, the heart's energy requirement is mainly furnished by provision of circulating carbohydrates and lipids through the coronary vasculature under normal aerobic conditions. The heart can also use its internal stores of energy, that is, glycogen and TAG, under certain circumstances, but this supply is quantitatively limited. However, current evidence suggests that most (~70%) of energy for heart is derived from the oxidation of fatty acids under physiological work load conditions [1, 6–8]. 

This conclusion has raised another important question. Because fatty acids are derived from both plasma albumin-bound nonesterified fatty acids (NEFAs) and plasma TAG-rich lipoproteins (TGRLPs), that is, VLDL and chylomicrons, what therefore is the relative contribution of each lipid source to the cardiac energy requirement? 

So far, many studies have been conducted to investigate the utilisation of NEFA (and to a lesser extent, chylomicrons) by heart. However, unlike these two FA sources, the true physiological role of VLDL within cardiac energy metabolism still remains uncertain: very few studies have been reported, and findings from these studies are somewhat discrepant. In this paper, we review these reports on the utilisation of VLDL by the myocardium, aiming to outline the possible role of VLDL in heart metabolism.

## 2. Can the Heart Function with VLDL?

Since the pioneering studies of Bing et al. [9], it has been fully appreciated that fatty acids are important substrates for myocardial energy requirements. However, it has not been clear what is the contribution of non-esterified fatty acid and esterified fatty acid to myocardial energy production. Even four decades later, it was still widely presumed that NEFA is the primary source of energy for the heart [8, 10], despite the fact that the molar concentration of fatty acid in TAG-rich lipoproteins may be up to an order of magnitude greater than that of albumin-bound NEFA [11]. 

However, in 1960, Ballard and colleagues suggested that it was actually esterified fatty acids which accounted for most of the total fatty acids extracted by the myocardium [12]. They found that during fasting more than 50% of fatty acids taken up by human hearts were derived from esterified fatty acids; in fasting dog, this figure was 77%. Importantly, this was the first experiment to show that the heart can utilise VLDL as a fuel because during fasting most of the esterified fatty acids are transported within the VLDL-TAG core.

Unfortunately, since then, although there have been some investigations on lipoprotein utilisation by heart [13, 14], compared to the multiple studies on cardiac non-esterified fatty acid metabolism, relatively few studies have examined this aspect of myocardial substrate selection. This is partly due to the difficulty in obtaining sufficient quantities of VLDL to test in an isolated heart system. In 2000, Bennett et al. [15] reported an extended liver perfusion technique to prepare species-specific radiolabelled VLDL. 

Following this technique, Hauton et al. investigated myocardial lipid substrate preference by comparing utilisation of NEFA, VLDL-TAG, and CM-TAG in isolated rat “working” hearts [10]. Cardiac mechanical function was maintained regardless of lipid substrate used. In other words, like NEFA and CM, VLDL is capable of supporting the heart energetically. However, some differences did exist: for example, the utilisation of VLDL-TAG by hearts was less than that of NEFA and CM-TAG. 

This is of particular interest. Mechanical performance of hearts perfused with VLDL as the sole source of lipid was comparable to those perfused with CM and/or NEFA, suggesting either that glucose oxidation is increased in VLDL perfused hearts, which has been shown not to be the case by our own work [16], or that “efficiency” of VLDL-TAG utilisation is greater than that of CM-TAG or NEFA (same work output for less substrate oxidised). If this is true, it could reasonably explain why under some energy stress conditions, such as diabetes [16] and sepsis [17], the utilisation and oxidation of VLDL are significantly increased. Indeed, in our previous work, there was evidence supporting this hypothesis: when the uptake pathway for VLDL was blocked, cardiac mechanical performance was severely affected [18], suggesting an important physiological role for this relatively small amount of lipid substrate assimilated by the heart.

These findings of significant myocardial VLDL utilisation were subsequently confirmed and extended [18]. Using radio-labelled VLDL particles from rat liver perfusions in isolated rat “working” heart preparations, we found that under normal physiological conditions, the myocardial uptake and utilisation of VLDL was less than that of CM, but mechanical function of the heart was comparable. Another significant difference was that only about half the VLDL-TAG assimilated by the heart was oxidized, whereas the proportion of CM-TAG oxidised reached 80%, indicating that the portion of TAG-rich lipoprotein-derived lipid channelled towards nonoxidative pathways (e.g., incorporation into tissue lipids) was protected. Considering that the heart can utilise carbohydrates, NEFA, CM, VLDL, amino acids, and so on as its fuel substrates under physiological conditions, VLDL probably functions differently to NEFA and CM in myocardial energy metabolism. Significantly, Hauton et al. [10] found that CM-TAG utilisation but not VLDL-TAG utilisation was suppressed in the presence of NEFA. However, TAG did not alter NEFA utilisation, suggesting that NEFA provides a significant contribution to the energy requirements of the working heart, whilst VLDL-derived lipid is preferentially required for nonoxidative metabolism.

However, there are studies challenging this conclusion. Elegant experiments by Goldberg's group utilising labelled TAG emulsions [11] suggested that fatty acid derived from the hydrolysis of TAG-rich lipoproteins may be the primary source of fatty acid utilised by the heart, particularly in the fed state. By using a new animal model with overexpression of anchored lipoprotein lipase (LPL) on the surface of cardiomyocytes (hLpL^GPI^ mice), Pillutla et al. [19] also found that VLDL isolated from fasting blood inhibited the uptake and oxidation of palmitate in the perfusate of both hLpL^GPI^ and wild-type hearts, suggesting that hearts can use TAG-derived fatty acids as an alternative fuel, and in some situations, as the primary source of fatty acid. Direct measurements of CM-TAG utilisation by mouse heart supports the importance of TAG-rich lipoproteins in cardiac energy provision [20, 21]. Furthermore, tissue-specific inhibition of cardiac TAG uptake by selective cardiac LPL knockout reveals both that the heart is a quantitatively important “sink” for plasma TAG [22] and that long-term cardiac function depends on adequate TAG provision (see below) [23].

Therefore, there is now little doubt that VLDL plays an important role in myocardial energetics and function, at least by acting as a metabolic substrate. But the difference in this aspect between VLDL and other lipid sources, such as CM and NEFA, could be of more important significance. Because its utilisation by the heart is relatively low but its efficiency is high, it is possible that VLDL serves as an alternative source of fatty acids for hearts under energy-stress conditions; however, under normal conditions, CM and NEFA are the major fatty acid (oxidative) sources for the heart whilst VLDL might play a regulatory role in myocardial lipid metabolism.

## 3. Is Lipoprotein Lipase or VLDL Receptor Responsible for the Uptake of VLDL by the Heart?

In 1943, Hahn reported that following heparin injection, the dog had decreased postprandial lipaemia [24], indicating that heparin caused the presence of a so-called lipid “clearing factor” in the circulation. This was the first work suggesting the role of lipoprotein lipase (LPL, E.C.3.1.1.34) in lipoprotein metabolism. It is now believed that LPL is the principal enzyme responsible for hydrolysis of TAG circulating within lipoproteins, making the fatty acids released available for use by subjacent tissues. 

There is strong evidence that heart expresses the LPL gene abundantly [25, 26]. Indeed, cardiac LPL activity alone (in the absence of LPL expression in other tissues) can normalise plasma TAG concentration in cardiac rescue LPL knockout mice [27]. LPL is synthesised by parenchymal heart cells, but requires subsequent transfer to the luminal surface of the endothelial cell, where it is bound to heparan sulphate proteoglycan (HSPG), in order to be physiologically active (functional). Hydrolysis of TAG by endothelium-bound LPL is widely regarded as the initial step for the “bulk” uptake of lipoprotein-TAG by peripheral tissues, including the heart [11, 22, 28–31]. Indeed, we found a significant decrease in the uptake and utilisation of VLDL-TAG (and CM-TAG) by the heart after LPL activity was inhibited [18], supporting a major role for LPL in mediating myocardial VLDL lipid assimilation, a conclusion supported by Cheng and Hauton who noted a correlation between cardiac LPL activity and TAG uptake during metabolic stress [32]. Interestingly, however, cardiac mechanical performance was not affected when VLDL utilisation was decreased [18], suggesting that there could be another mechanism for the heart to take up VLDL, and this small pool of VLDL is effective in maintaining the heart in good working condition even when LPL-mediated hydrolysis is blocked.

It should be noted that LPL-mediated hydrolysis of lipoproteins can occur in the plasma following LPL release from the endothelial cell surface by heparin; hence, Hahn found a rapid clearance of blood lipid after injection of heparin [24]. Moreover, some LPL probably dissociate from the endothelial cell in association with the lipoprotein particle during lipolysis and continues to lipolyse TAG-rich lipoproteins in the bloodstream. But it is still not clear how much lipolysis occurs on the cell surface compared to the plasma [30], and what the relative contribution of these two sources of lipolysis is to cardiac energy provision. 

Although LPL is also present on the surface of cardiomyocytes, it is generally believed that the luminal surface of the endothelial cell is the site where hydrolysis of the lipoprotein-TAG core take place. However, by using a transgenic mouse model with an LPL transgene construct expressed and anchored on the cardiomyocyte surface, Yagyu et al. [33] found that this pool of LPL was responsible for the increased uptake of TAG by the heart. Furthermore, a new model of cardiomyopathy was created due to the excessive myocardial uptake of TAG via LPL action, suggesting that cardiomyocyte cell surface LPL is physiologically functional in terms of mediation of cardiac TAG uptake. 

As reactant and product of the lipolytic process, respectively, TAG and free fatty acid have different effects on LPL activity. Circulating TAG increased LPL activity at the coronary lumen but decreased it on the cardiomyocyte cell surface, suggesting that TAG facilitates LPL translocation from cardiomyocyte to the myocardial endothelial lining [34], and we found that VLDL was capable of modulating LPL translocation in isolated hearts [10, 18]. However, Anderson et al. [35] found that free fatty acids selectively decreased heparin-releasable LPL activity in cultured cardiomyocytes, possibly through end-product inhibition of lipolytic processes, and despite an increase in immunodetectable LPL on the cardiomyocyte cell surface. 

All these studies suggest that LPL is responsible for the uptake of VLDL-TAG by the heart. However, other factors may be important in VLDL assimilation. 

In 1967, Enser et al. [14] used ^14^C labelled chyle lipids to perfuse isolated rat hearts. They found that at least part of the ^14^CO_2_ production could have been derived from the oxidation of ^14^C labelled free fatty acid produced in the perfusion medium via the function of the so-called “clearing-factor lipase”. They also found that the whole lipid particle was being taken up and oxidised by the heart. These results suggested that TAG-rich lipoprotein could both be hydrolysed to produce free fatty acid outside the heart and could also be taken up by the heart as a whole particle. To our knowledge, this was the first work to indicate that besides the LPL-mediated hydrolysis there is an alternative mechanism for hearts to assimilate TAG-rich lipoproteins. It is now believed that this pathway is receptor-mediated endocytosis. 

At least two kinds of lipoprotein receptor may be important in the myocardium in this respect: the VLDL receptor [36–39] and the apo-B48 receptor [40], both of which have been found in abundance in those tissues active in fatty acid utilisation, including heart, suggesting a potential role in delivery of fatty acid to these tissues for metabolic purposes.

The VLDL receptor (VLDL-R) is a 118-kDa protein and a member of the expanding mammalian low density lipoprotein receptor (LDL-R) gene family with ligand specificity: it binds apo-E but not apo-B. Notwithstanding some species differences in tissue distribution of VLDL-R [41], it is reasonable to believe that this receptor is responsible for the delivery of VLDL particles into the heart. Indeed, since VLDL-R cDNA was cloned in 1992 [42], many studies have been performed to identify the function(s) of this receptor and findings are comparable, which suggests a relationship between myocardial VLDL-R expression and VLDL uptake.

For example, VLDL-R protein expression in heart was found to increase progressively with fasting [43], suggesting its potential role of delivering VLDL-TAG to the myocardium since VLDL production is increased in fasting. In addition, in hypertrophic hearts, VLDL-R mRNA decreased [44]; the decreased VLDL-R expression could be linked to the energy substrate switch from lipid to glucose known to occur in myocardial hypertrophy [45, 46]. Kamataki et al. also found that in the Balb/c fasting mice, VLDL-R as well as other molecules involved in cardiac lipid metabolism, including LPL, increased [47]. This is probably a mechanism for heart to adapt to differing energetic situations. More recently, Zenimaru et al. [48] found that glucose deprivation from the culture medium significantly induced AMPK activation, followed by increased VLDL-R expression and hence increased uptake of VLDL in rat L6 myoblasts. This is interesting because it suggests that the heart tends to utilise the more “efficient” VLDL via regulation of VLDL-R expression to deal with the substrate switch from glucose to lipid utilisation which is known to occur in conditions such as fasting, sepsis, and diabetes [45].

However, we used suramin to block lipoprotein receptors on the cardiomyocyte surface [18] and demonstrated some interesting findings. Firstly, VLDL-TAG fatty acid uptake was not affected during receptor blockade but VLDL particle uptake was inhibited (confirmed by decreased VLDL-cholesterol uptake by the heart); secondly, the oxidation rate of VLDL-TAG significantly decreased and the proportion of TAG oxidised also decreased; thirdly, cardiac mechanical function was significantly impaired following lipoprotein receptor blockade [18]. Given that myocardial VLDL-TAG uptake and utilisation, but not cardiac mechanical function, decreased when LPL was inhibited, these results suggest that, although quantitatively less important than LPL-mediated hydrolysis, VLDL receptor-mediated uptake is a functionally critical mechanism for the heart to effectively utilise VLDL. Furthermore, receptor-mediated TAG uptake is preferentially directed towards oxidation rather than esterification, suggesting a correlation between lipid uptake mechanisms and metabolic “channeling” within the cardiomyocyte [18].

Unlike the VLDL receptor, the apo-B48 receptor is an apo-E- and LPL-independent membrane protein originally found to be responsible for the uptake of TGRLP by reticuloendothelial system (and hence also termed the TGRLP receptor), and believed to deliver lipid and lipid-soluble vitamins to monocyte-macrophages for nutritional purposes under physiological conditions [49]. However, little is known about its role in cardiac lipid metabolism. Clues are that peroxisome proliferator-activated receptor (PPAR)-*α* and PPAR*γ* activators significantly suppress the expression of apo-B48 receptor mRNA in human monocyte-macrophages; moreover, these activators also inhibit the expression of the apo-B48 receptor protein and; hence, the receptor-mediated lipid accumulation of TGRLP by monocytes *in vitro* [50, 51]. Because the heart expresses PPAR*α*, PPAR*γ*, and apo-B48 receptor (albeit the last at relatively low level), it is possible that the apo-B48 receptor could also be involved in cardiac lipid metabolism. 

Clearly, both LPL and lipoprotein receptors (including the VLDL receptor) are responsible for the uptake of VLDL-TAG (as well as chylomicrons remnants [52]) by the heart—but several lines of evidence suggest that interactions between the two mechanisms exist. There are at least three putative mechanisms of interplay between LPL and VLDL-R [37, 38]: (1) as a ligand, LPL can directly bind to the receptor; (2) acting as a molecular “bridge”, LPL facilitates the binding of VLDL particles to heparan sulphate proteoglycans prior to interaction with the receptor [38]; (3) LPL converts VLDL particles to smaller remnants (intermediate density lipoprotein (IDL)), which can subsequently be endocytosed by the receptor (see [53]).

However, interactions between VLDL-R and LPL could work in a reverse fashion: the VLDL receptor acts as an “anchor” to facilitate hydrolysis of TAG-rich lipoproteins by LPL [54]. Indeed, VLDL-R-deficient mice have reduced LPL activity because the VLDL receptor is involved in the translocation of LPL from cardiomyocytes to the endothelial surface [55], causing hypertriglyceridaemia associated with reduced catabolism of VLDL-TAG by LPL. Further strong support for this mechanism is that Receptor-Associated Protein (RAP), a 39-kDa protein that inhibits both the LDL receptor-related protein and the VLDL receptor, also inhibits LPL activity in mice expressing VLDL-R, but RAP is less effective in VLDL-R knockout animals [55]. The effect of RAP on LPL is presumably therefore at least partly via its inhibition of VLDL-R, which again supports the concept of the regulation of LPL by VLDL-R.

Although the two mechanisms are responsible for the uptake of VLDL by the heart, under physiological conditions, myocardial utilisation of VLDL is relatively low [10, 18]. This suggests that compared to other lipid substrates such as NEFA and CM, VLDL does not act as a major energy source for hearts in this situation. Therefore, what is the true physiological role of VLDL? Because it is the ligand for both LPL and VLDL-R, VLDL itself could be a “bridge” connecting the two proteins and act as an important regulator in cardiac energy metabolism; also, because VLDL-TAG core is a more “efficient” lipid substrate for the heart [10, 16, 18] it could be an alternative or “external” energy storage for hearts to utilise in order to adapt to various energy-stress conditions, such as diabetes.

## 4. How Does VLDL Utilisation by the Heart Change in Diabetes?

In diabetes, downregulated glucose transport and metabolism as the consequence of insulin deficiency (type 1) and resistance (type 2), result in fatty acid oxidation becoming the almost exclusive energy source for the heart [45, 56, 57]. Little is known about TAG metabolism in the diabetic heart, but investigation of myocardial utilisation of VLDL in diabetes could provide some clues about the role of this particle in cardiac energy metabolism. To date, the findings relating to cardiac TAG metabolism in diabetes are extremely discrepant, making it difficult to discern the true role of VLDL, and this may be a reflection of widely varying models of disease severity and length of exposure [58]. Both decreased and increased VLDL utilisation by the heart have been reported. Interestingly, however, the two apparently incompatible findings can each explain two widely accepted features of diabetes: hypertriglyceridaemia and diabetic cardiomyopathy (a severe cardiovascular complication due to “lipotoxicity” rather than other cardiac risk factors, such as hypertension and coronary atherosclerosis).

Decreased cardiac LPL activity in diabetic rodent hearts has been reported [59, 60]. In 1983, O'Looney et al. [60] reported decreased VLDL metabolism by streptozotocin (STZs) induced diabetic hearts, associated with decreased heparin-releasable LPL activity, both effects were completely reversed by the administration of insulin. More recently, cardiac VLDL-R protein levels, as well as postheparin LPL activity, were found to decline significantly in STZ-induced diabetes [61]. Yagyu et al. [55] also found that disruption of VLDL-R resulted in hypertriglyceridaemia associated with decreased LPL activity in mice. In addition, Kobayashi et al. [59] observed decreased postheparin LPL activity in db/db mice, a type 2 diabetic animal model. These results could explain diabetic hypertriglyceridaemia; additionally, in support of this mechanism, Chen et al. found that hypertriglyceridaemia in STZ rats was not primarily due to VLDL overproduction in liver but to a peripheral VLDL-TAG removal defect [62]: since LPL and VLDL-R are both down-regulated in diabetes, VLDL particles accumulate in the plasma, resulting in hypertriglyceridaemia. 

However, decreased LPL activity and VLDL-R deficiency in diabetes cannot explain the increased fatty acid oxidation observed in these hearts, which is another characteristic feature of the diabetic state. In contrast to the findings reported above, meticulous research conducted in Rodrigues' group demonstrated increased cardiac heparin-releasable LPL activity in STZ-diabetic [58] and SHR-diabetic rats [63], which they hypothesised could increase fatty acid supply to the heart. Subsequent studies from the same group confirmed the hypothesis in this model: increased myocardial VLDL metabolism associated with elevated heparin-releasable LPL activity in STZ-induced moderately diabetic rat hearts was reported [64]. The authors believed that the rapid removal of VLDL-TAG by diabetic heart could be one mechanism for the increased provision of fatty acid for energy production, to compensate for the diminished contribution of glucose as an energy source. Furthermore, this mechanism could be responsible for the development of diabetic cardiomyopathy due to excess delivery of potentially toxic fatty acid to the heart (“lipotoxicity”). Intracellular lipids accumulate and can, either by themselves or via production of second messengers such as ceramides, provoke cell death [25, 65].

Our studies support Rodrigues's findings. We found that cardiac heparin-releasable LPL activity increased in both type 1 [16] and type 2 diabetic rats [66], and VLDL-TAG oxidation increased significantly in diabetic hearts. In addition, when hearts from both control and diabetic rats were perfused with VLDL particles prepared from STZ-treated animals, heparin-releasable LPL activity increased, suggesting that diabetic VLDL particles may stimulate LPL activity, and indicating a possible regulatory role for VLDL. Consistent with this, we have also found significant intracellular TAG accumulation, derived from exogenous VLDL, in both types of diabetes regardless of unimpaired cardiac function at an early stage of the disease [16, 66]. Further evidence of a role for VLDL as a metabolic signal and cellular regulator has recently been provided by the demonstration that VLDL modulates myocardial SERCA-2 expression and calcium handling, an effect potentiated by hypoxia [67].

One explanation for the variable and condition-dependent nature of VLDL action and metabolism may be the complex nature (and potentially key importance) of the composition of the VLDL particle itself. VLDL particles are composed of proteins and lipids. The apoprotein components maintain the structure of the particles but also have regulatory and signalling functions [68]; lipid constituents include TAG, phospholipids (PL), cholesterol, cholesterol ester, and free fatty acids, with potentially complex surface interactions with target routes. Furthermore, dynamic alterations in the composition of VLDL particles occur physiologically as well as pathologically, and this can profoundly affect their efficacy as a target and substrate for both LPL and VLDL-R. Upon its entry into the bloodstream, VLDL acquires apo-C and apo-E by transfer from circulating plasma high-density lipoproteins (HDL). Subsequently, the TAG core of VLDL is hydrolysed by lipoprotein lipase (LPL), resulting in free fatty acid release and formation of smaller remnant particles, that is, intermediate-density lipoprotein (IDL), and ultimately low-density lipoprotein (LDL) which are enriched in cholesterol. The remnant particles are cleared from the plasma by receptor-mediated endocytosis [69]. Any compositional alteration is of great potential significance regarding metabolism of the lipoprotein particle (and its remnant) under pathophysiological conditions. For example, apo-E is an important ligand of VLDL-R [36, 39] and can anchor apo-E-rich lipoproteins to the HSPG site of the cells [30]. Apo-CII is an activator of LPL, whereas apo-CIII inhibits the activity of the enzyme [30, 36, 39]. Several studies have analysed the composition of VLDL particles in diabetes [70, 71]. Again, however, the results are discrepant. Increased [72], unchanged [73], and decreased [16, 74] TAG contents in diabetic VLDL have all been reported. However, the particle is complex and subtle differences in lipid or apoprotein makeup may have a major impact on subsequent particle efficacy and hence action, and this may in part account for the variable findings. Thus, Sambandam et al. [64] found increased myocardial VLDL metabolism in type 1 diabetes but also reported that there was no apparent difference in apolipoprotein composition in VLDL particles obtained from control and diabetic rats; the authors considered that VLDL prepared from diabetic rats is a normal substrate for LPL. This is in contrast to our own findings [16] (as well as O'Looney's earlier work [71]) although both of these studies also demonstrated increased VLDL metabolism in STZ-induced diabetes. Firstly, we found that the TAG : PL ratio of VLDL particles from diabetic rats was significantly lower compared to control VLDL, suggesting that VLDL particles in type 1 diabetes are smaller (since TAG : PL ratio approximates to core-to-surface ratio and, thus, lipoprotein particle size [75]). If this is the case, VLDL particles would interact less effectively with LPL. Secondly, apo-E content was increased in diabetic VLDL particles. This is probably a mechanism for VLDL particles to adapt to diabetic conditions since increased apo-E content facilitates the interaction between VLDL and VLDL-R, and hence also between VLDL and LPL. By contrast, Ishibashi et al. [73] reported no change in VLDL lipid content but they found that apo-CIII/CII ratio increased in diabetes, suggesting a factor contributing to hypertriglyceridaemia since apo-CIII is an inhibitor of LPL.

Hence, research to date suggests that VLDL is indeed a key factor in the pathogenesis of hypertriglyceridaemia and cardiac dysfunction in diabetes, although its precise role is yet to be fully elucidated. Meanwhile, evidence remains conflicting; for example, in human type 1 diabetic patients VLDL metabolism has been reported as normal [72]. Furthermore, Sambandam et al. [64] challenged others' works by demonstrating that elevated TAG levels found in STZ-diabetic rats was abolished after 16-hour fasting, suggesting that increased chylomicron-TAG may contribute to the diabetic hypertriglyceridaemia: like VLDL, chylomicrons are abnormal in composition in diabetes [70, 76] and their normally rapid peripheral tissue clearance may therefore also be delayed in this state. Such a mechanism would reconcile the coexistence of hypertriglyceridaemia (CM) and increased myocardial TAG-FA metabolism (VLDL) mediated through LPL and VLDL-R uptake mechanisms.

Interestingly, therefore, changes in myocardial VLDL utilisation in diabetes can explain the two key features of this metabolic disorder: hypertriglyceridaemia and diabetic cardiomyopathy.

## 5. Is VLDL an Extracardiac Energy Reserve?

By using VLDL particles prepared from perfused livers from control and diabetic rats, studies to investigate myocardial VLDL utilisation under normal conditions and in various metabolic disease states have been conducted in our lab [16, 18, 66]. From these studies it has emerged that one possible role of VLDL is to act as an exogenous (extratissue) energy storage or buffer for the heart. Unlike endogenous myocardial energy storage, that is the intramyocellular pool of TAG and glycogen, plasma VLDL is far more quantitatively significant. Furthermore, since it is delivered by the blood, it will be accompanied by oxygen, absolutely required for oxidation of TAG-derived fatty acid. Therefore, the heart mobilises this pool of TAG to adapt itself to specific energetic-stress conditions, for example, diabetes and sepsis. Conversely, the concept of plasma VLDL as a cardiac TAG buffer is supported by the observation of lipoprotein secreting capacity by the heart [77], permitting two-way interchange of TAG/lipoprotein between cardiomyocyte and plasma. Such a mechanism would provide the heart with a mechanism to prevent lipid overload and hence lipotoxicity.

The key evidence for this is that under physiological conditions only about half of VLDL-TAG assimilated was oxidised in the cardiomyocyte [18], and the oxidation rate of VLDL-TAG was markedly lower than that of CM-TAG [10, 18] and NEFA [10]. This suggests that the primary purpose of VLDL delivery to the heart may not be for oxidation to produce ATP (although the heart did work well whilst oxidising some of the VLDL-TAG assimilated [10, 18]) under these physiological conditions. By contrast, more than 80% of VLDL-TAG assimilated was oxidised in type 1 [16] and type 2 [66] diabetes, and the oxidation rate was also significantly higher than that under physiological workload. Furthermore, we have demonstrated that cardiac VLDL metabolism and functional efficacy is significantly increased in sepsis/endotoxinaemia [17]; VLDL is a better cardiac substrate in endotoxinaemia, and this is a function of some change in the composition of the “endotoxic” VLDL particle rather than the heart itself, since the effect depends on the endotoxic state of the VLDL and not on the endotoxic state of the myocardium. This finding was supported by the demonstration of altered composition of the VLDL particle secreted by the endotoxic liver [17, 78, 79]. The primary importance of the VLDL particle *per se* in determining its function and fate is emphasised by the recent observation that cardiac VLDL-R is downregulated in sepsis/endotoxinaemia [80]—hence, alterations in VLDL composition are essential determinants in its utilisation, irrespective of specific uptake route (indeed “endotoxic” VLDL secreted during sepsis has been suggested as a vital component of the host immune response [81, 82]). Again, this argues in favour of a vital role for VLDL in energy provision in metabolic stress states, a notion supported by the observation of increased hepatic VLDL synthesis and secretion in both sepsis [83–86] and diabetes [70, 87–89]. If VLDL does provide an energy reserve for the heart, it is understandable that myocardial VLDL-R expression is increased during the physiological conditions of development [39] and fasting [43, 47]: the heart uses this energy storage to adapt itself more efficiently to changing circumstances when undergoing switching of energy substrates. 

These findings indicate that VLDL is mobilised under specified pathophysiological conditions as a major myocardial energy source to provide ATP for maintenance of cardiac function.

## 6. Summary

The role of VLDL, a TAG-rich lipoprotein, in myocardial energy metabolism has yet to be fully elucidated. Unlike NEFA and CM, VLDL is not a preferred substrate for the heart under physiological conditions. However, the heart utilises more VLDL for the production of ATP in diabetes and sepsis/endotoxinaemia. Also, under other conditions such as fasting and development, when energy substrate switching occurs, VLDL appears to be an easily accessed energy source for the heart. Two possible roles of VLDL in terms of cardiac energy metabolism have been suggested: a “metabolic regulator” particularly for LPL activity, and “exogenous energy storage” for hearts. Further investigations are required to confirm these roles of the VLDL particle.
